# Effects of separated pair housing of female C57BL/6JRj mice on well-being

**DOI:** 10.1038/s41598-022-12846-6

**Published:** 2022-05-25

**Authors:** K. Hohlbaum, R. Merle, S. Frahm, A. Rex, R. Palme, C. Thöne-Reineke, K. Ullmann

**Affiliations:** 1 Institute of Animal Welfare, Animal Behavior, and Laboratory Animal Science, Department of Veterinary Medicine, Freie Universität Berlin, Berlin, Germany; 2 Institute for Veterinary Epidemiology and Biostatistics, Department of Veterinary Medicine, Freie Universität Berlin, Berlin, Germany; 3 Stem Cell, Technology Platform, MDC Berlin-Buch, Berlin, Germany; 4 Department of Experimental Neurology, Charité - Universitätsmedizin Berlin, Berlin, Germany; 5 Unit of Physiology, Pathophysiology and Experimental Endocrinology, Department of Biomedical Sciences, University of Veterinary Medicine, Vienna, Austria; 6 Research Facilities for Experimental Medicine, Charité - Universitätsmedizin Berlin, Berlin, Germany; 7 Nuvisan ICB GmbH, Berlin, Germany

**Keywords:** Animal behaviour, Animal physiology, Zoology

## Abstract

In laboratory animal facilities, it is a common code of practice to house female mice in groups. However, some experimental conditions require to house them individually, even though social isolation may impair their well-being. Therefore, we introduced a separated pair housing system and investigated whether it can refine single housing of adult female C57BL/6JRj mice. Individually ventilated cages (IVC) were divided by perforated transparent walls to separate two mice within a cage. The cage divider allowed visual, acoustic, and olfactory contact between the mice but prevented interindividual body-contact or food sharing. Short- and long-term effects of the separated pair housing system on the well-being of the mice were compared with single and group housing using a range of behavioral and physiological parameters: Nest building behavior was assessed based on the complexity of nests, the burrowing performance was measured by the amount of food pellets removed from a bottle, and trait anxiety-related behavior was tested in the free exploratory paradigm. For the evaluation of the ease of handling, interaction with the experimenter's hand was monitored. Social interaction with unknown conspecifics and locomotor activity were investigated in a test arena. Moreover, body weight and stress hormone (metabolites) were measured in feces and hair. After the mice spent a day under the respective housing conditions, concentrations of fecal corticosterone metabolites were higher in separated pair-housed mice, and they built nests of a higher complexity when compared to single-housed mice. The latter effect was still observable eight weeks later. In week 8, separated pair-housed mice showed less locomotor activity in the social interaction arena compared to mice from the other housing systems, i.e., single and group housing. Regardless of the time of testing, pair housing improved the burrowing performance. Separated pair-housed mice were more difficult to catch than group-housed mice. Hair corticosterone, progesterone, and dehydroepiandrosterone concentrations changed with increasing age independently of the housing system. There were no effects of the housing systems on trait anxiety-related behavior in the free exploratory paradigm, voluntary interaction with the experimenter’s hand, and body weight. Overall, the transfer to the separated pair housing system caused short-term stress responses in female C57BL/6JRj mice. Long-term effects of separated pair housing were ambiguous. On one hand, separated pair housing increased nesting and burrowing behavior and may therefore be beneficial compared to single housing. But on the other hand, locomotor activity decreased. The study underlined that the effects of the housing conditions on physiological and behavioral parameters should be considered when analyzing and reporting animal experiments.

## Introduction

In natural habitats, female mice live in polygamous family groups as breeding or subordinate females supporting the rearing of other offspring^1^. In laboratory animal facilities, female mice are usually housed in breeding groups with one male or in single-sex-groups to prevent uncontrolled breeding^2–4^. However, some experimental settings (e.g., calorimetric measurements, monitoring of individual behaviors, some surgical procedures) do not allow group housing and require individual housing, which may influence both animal welfare and scientific data. Although single housing is not as widely studied in females as in males, the literature reveals that it is associated with diverse behavioral and biochemical consequences, which depend on the age of the animals (i.e., after weaning, during adolescence or adulthood) and the duration of social isolation (i.e., some days to many weeks)^5,6^.

Inconsistent findings were made according to exploratory and anxiety-related behavior. Kulesskaya et al.^7^ analyzed effects of nesting material and social isolation on the behavior of female C57BL/6JOlaHsd mice for two months starting at the age of three weeks. They found enhanced exploratory behavior and stress tolerance for single-housed females in the elevated plus maze, light–dark test, and Morris water maze^7^. Further studies by Palanza et al. analyzed sex- and estrous cycle-related as well as social isolation aspects with regards to social dominance, depression- and anxiety-like behavior in mouse models^8,9^. In contrast to Kulesskaya et al.^7^ increased anxiety and reduced exploration were shown in the open field in isolated CD-1 Swiss female mice, which had been isolated at 90 days of age for one week^8,9^. Arndt et al. focused on the impact of individual housing on behavior and stress responses in C57BL/6 and BALB/c mice of both sexes^10^. Unlike Kulesskaya et al.^7^ isolation associated changes in anxiety-like behavior were not identified in C57BL/6 females and only risk assessment was elevated in single-housed BALB/c females^10^. These findings were supported by Bailoo et al.^11^ The authors investigated the effects of isolated housing and weaning age on the phenotype of male and female RjORL:SWISS mice that had been single-housed at the age of 7–8 weeks for two months^11^. No effects on body weight, fecal glucocorticoid metabolites, stereotypic behavior and anxiety-related behavior were identified^11^. When Bailoo et al. described the effects of single housing, it must be taken into consideration that social isolation of rodents is used as a model for psychopathological disorders like anxiety and depression^8,12,13^. Besides the described alterations of behavior, social isolation is also accompanied with changes in the brain structure^14,15^ and gene expression^6^. In 15–17 week old adult male and female C57BL/6 J mice, 8-week social isolation resulted in alterations of hippocampal neurons as well as neuroplasticity and a decreased memory acquisition, consolidation, and retrieval^14^. Furthermore, post weaning social isolation for four weeks was compared between male and female mice. Females but not males showed reduced serotonergic fiber density^15^. Feeding behavior was investigated in 6–10 week and 79–91 week old male and female C57BL/6 mice after two weeks of isolation. Isolation increased hypothalamic preproghrelin gene expression in young females accompanied with higher food intake^6^. The authors of these studies associated structural and genetic brain alteration at least partly with the behavioral changes.

To reduce social isolation for mice that must be individually housed, we established the separated pair housing system, where individually ventilated cages (IVC) were divided by a transparent, perforated wall^16^. The wall prevented physical contact but enabled olfactory, visual, and auditory interactions between two mice^16^. The aim was to refine conventional single housing by allowing at least a certain degree of sensory contact^16^. We could previously demonstrate positive long-term effects on nesting and burrowing behavior when housing male C57BL/6JRj as separated pairs^16^. However, short-term separated male:male pair housing was shown to affect heart rate, body temperature, motor activity, body weight, and nest building behavior in 8–9-month-old vasectomized Hsd:NMRI mice, indicating impaired well-being^17^.

In this study, we investigated whether short-term and long-term separated pair housing for one day and eight weeks, respectively, can be used to refine conventional single housing of adult female C57Bl/6JRj mice. Separated pair housing was compared with single and group housing. We systematically analyzed a range of behavioral parameters, such as nesting, burrowing behavior, anxiety-related behavior, ease of handling, and social interaction. Moreover, body weight and stress hormone (metabolites) concentrations were measured and compared between separated pair housing, single housing, and group housing.

## Material and methods

The first part of the study, i.e., separated pair housing of male mice, has already been published. Material and methods of the second part of the study, i.e., separated pair housing in female mice, are partly identical^16^.

### Ethics

All animal experimentation was approved by the Berlin State Authority and the Ethics committee (“Landesamt für Gesundheit und Soziales”, permit number: G 0251/18)^16^. It was registered in the Animal Study Registry (https://doi.org/10.17590/asr.0000101)^16^. The study was performed according to the German Animal Welfare Act, the Directive 2010/63/EU for the protection of animals used for scientific purposes, and the Charité Animal Welfare guidelines^16,18^.

Federation of European Laboratory Animal Science Associations (FELASA) guidelines and recommendations for the care and use of laboratory animals were observed^16^. We followed the recommendations of the ARRIVE guidelines^19^. Humane endpoints for group-housed mice were injuries, i.e., wounds associated to fights between the cage mates. Fight associated wounds are usually found at the back, tail, genital region, or even in the face. Moreover, humane endpoints were defined for target animals in the social interaction test: mice were not used as target mice if they did not acclimatize to the perforated polycarbonate box (e.g., biting the bars of box, persistent restlessness, stereotypies).

Sample size calculation (primary outcome measure: effect of the housing systems on hair corticosterone) was performed using package “pwr” in R (power of 80%, standardized effect size of 0.5)^16^.

Data supporting the findings of this study are available within the supplementary table.

### Animals

A total number of 60 adult female C57BL/6JRj mice were obtained from Janvier Labs (Saint-Berthevin Cedex, France) at 4 weeks of age. The acclimatization period to our animal facility was three weeks^16^. We chose this strain because it is among the most used strain in animal-based research. The mice were assigned to the following study groups by simple randomization: single housing (*n* = 16), group housing (*n* = 16), and separated pair housing (*n* = 16)^16^. Twelve mice served as target animals in the social interaction test^16^. Ear punches were used for identification of the animals.

### Housing conditions

Mice were housed in individually ventilated cages (IVCs) containing wooden bedding material (SafeR Select, Safe, Augy, France), nestlets (Ancare, UK agents, Lillico, United Kingdom), and a red, triangular plastic house (length: 12,5 cm, width: 11 cm, height: 6 cm; Tecniplast, Italy) or a plastic tunnel (length: 10 cm, diameter: 4,5 cm, in-house fabrication)^16^. The animals were maintained under standard conditions (room temperature: 22 ± 2 °C; relative humidity: 55 ± 10%) on a light:dark cycle of 12:12 h of artificial light (lights on from 6AM to 6PM)^16^. They had free access to water and were fed pelleted mouse diet ad libitum (V1534-000, Ssniff, Soest, Germany)^16^.

**Figure 1:**
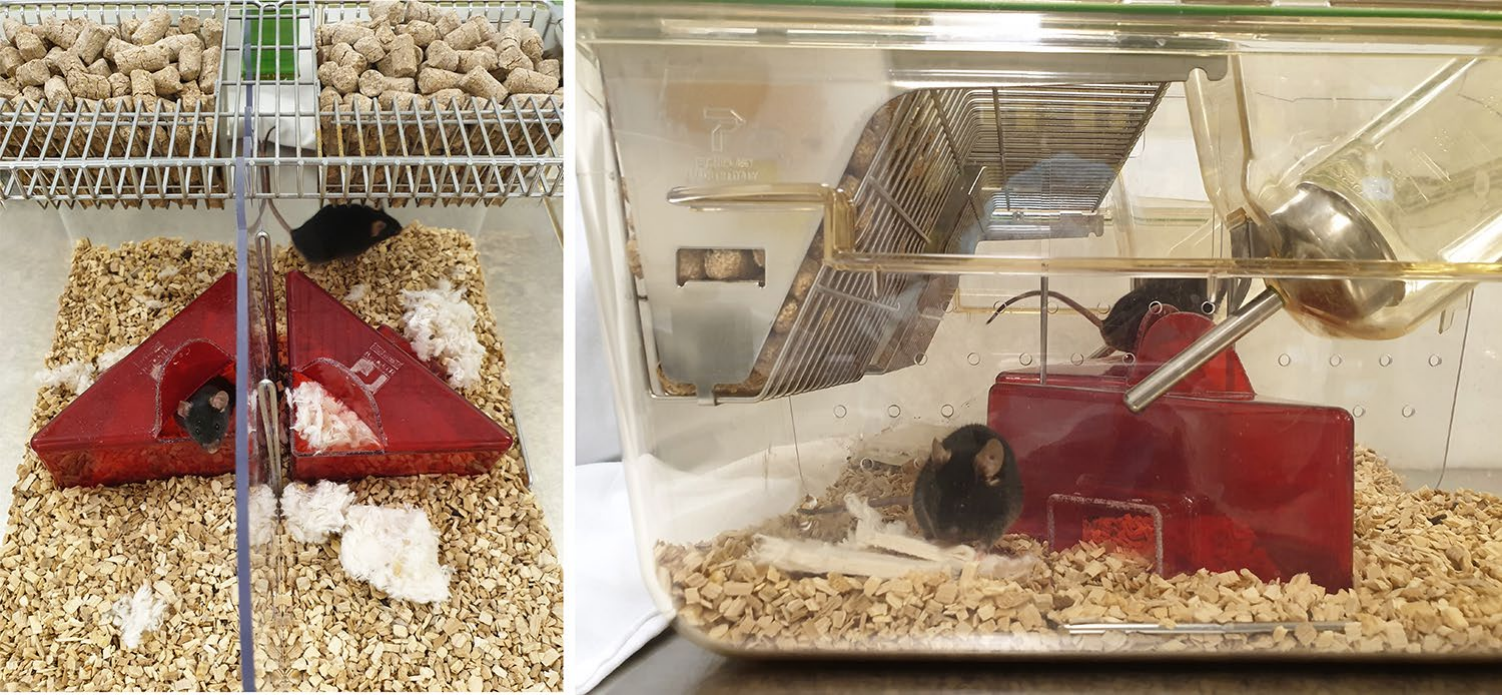
Pair housing system from the top and side view.

During acclimatization, mice were kept in Polysulfone type II long cages (544 cm^2^ (32 cm × 17 cm) × 13 cm) in groups of 3–4 siblings per cage^16^. After the acclimatization period, mice were assigned to one of the three study groups. In contrast to animals assigned to the study groups “single housing” and “pair housing”, the subset of mice assigned to "group housing" were kept in the same social groups assigned during acclimation^16^. Single-housed mice were transferred to Polysulfone type I long cages (416 cm^2^ (32 cm × 13 cm) × 13 cm) and separated pair-housed mice to Green Line IVC Sealsafe PLUS Rat GR 900^16^. The latter had no contact with each other before, i.e., they were unfamiliar to each other^16^. The separated pair-housing system was divided into two compartments (size of each compartment: 527 cm^2^ (31 cm × 17 cm) × 15 cm) by a perforated transparent wall (Fig. 1)^16^. The cage divider allowed olfactory, acoustic, and visual communication^16^. Independent of the study group, all animals were individually tested according to the following testing schedule.

### Testing schedule

**Figure 2:**
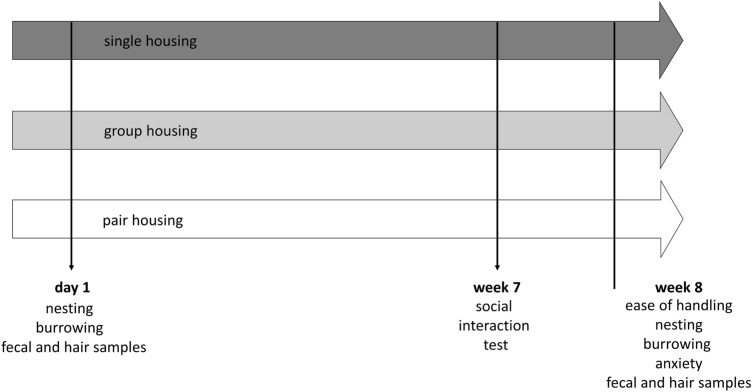
Testing schedule. Indicators of well-being were measured in single-, group-, and pair-housed mice on day 1, week 7, and week 8 after transfer to the indicated housing systems.

Figure 2 illustrates the testing schedule. After a 3-week habituation period to the animal facility, the mice were transferred to their respective housing system and hair samples were collected^16^.

**Figure 3:**
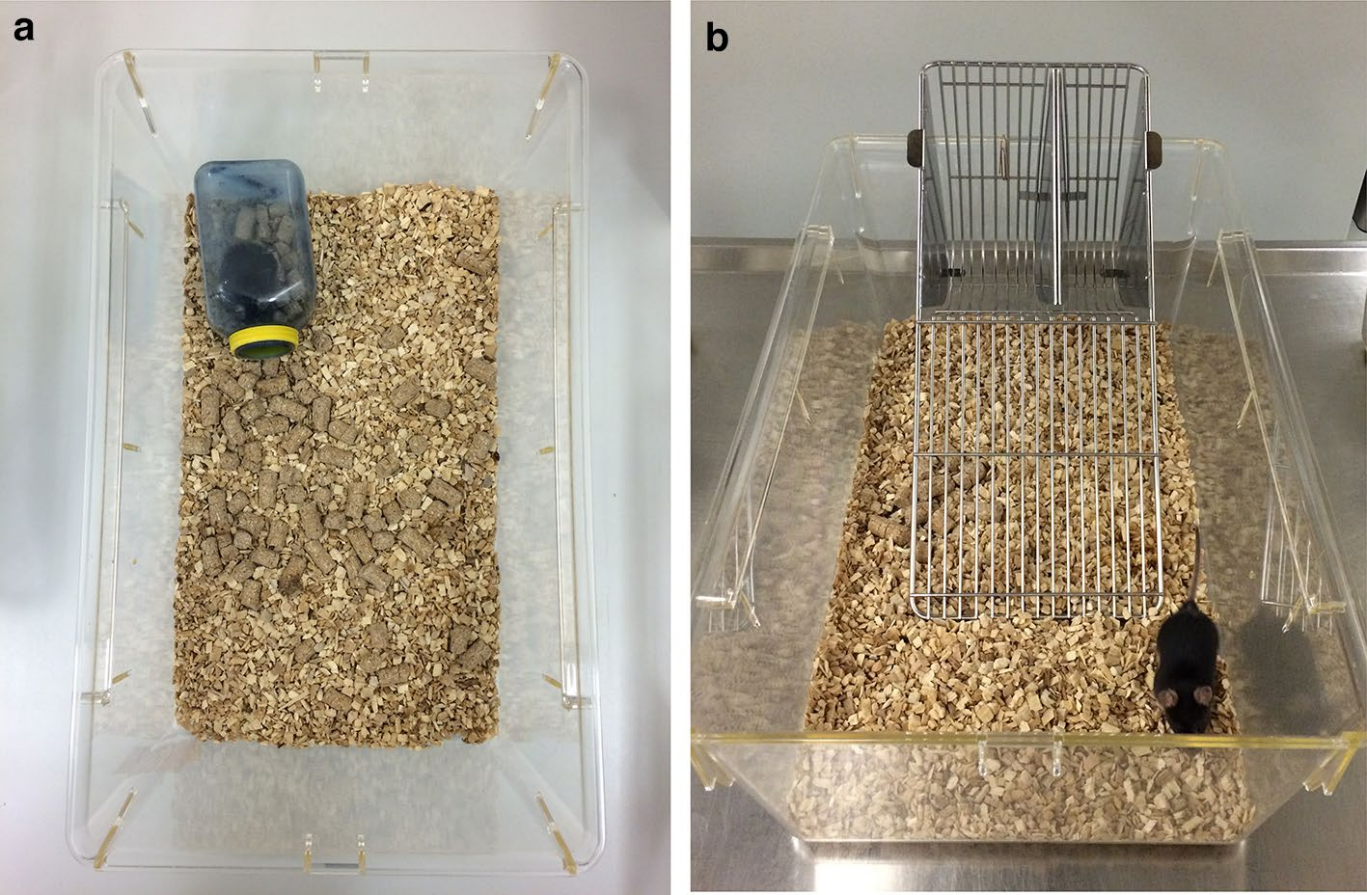
Testing cage. A Polysulfone type III cage was used to test burrowing behavior (**a**) and trait anxiety-related behavior in the free exploratory paradigm (**b**). Note that the gridded cage lid with water bottle and food pellets was removed for the image (**a**). When the mice habituated to the testing cage and burrowing behavior was monitored, the cage was closed by a gridded lid. For the free exploratory paradigm, the lid was removed.

In the morning of the following day, the nests built in the home cages were scored between 7.00 and 7.30 AM. Then the animals were moved in their home cages to the testing room and allowed to acclimatize (in their home cages) to the new environment for approximately an hour (~ 7.30–8.30)^16^. The mice were individually transferred from their home cages to a testing cage (Polysulfone type III; Fig. 3), where they habituated for another 30 min (~ 8.30–9.00)^16^. Burrowing was assessed over a 2-h period (~ 9.00–11.00). The animals were then transferred back from the testing cage to their home cage. Finally, fecal pellets were collected from the testing cage^16^. In week 7, the social interaction test was carried out. In week 8, the same schedule as described for day 1 was followed and, additionally, trait anxiety-related behavior was investigated using free exploratory paradigm in the same testing cages after the burrow had been removed^16^. Mice were handled and weighed during routine cage changes (i.e., once a week), and in week 8 the ease of handling was evaluated^16^.

Experimenters were blinded wherever possible (burrowing, anxiety-related behavior, social interaction, hormone (metabolites) analysis)^16^. However, the study design did not allow to blind experimenters for nest scoring and evaluation of the ease of handling because these tests were performed in the home cages of the animals, which were easily recognizable^16^.

### Nesting

A day before the nests were scored, cages were routinely changed^16^. A square cotton nestlet of 2.4–2.6 g (Ancare, Bellmore, NY, USA) was placed in the front left corner^16^. To prevent the mice from building their nests in a shelter, houses or tunnels were added to the cages after the nests had been scored in the morning of the following day (i.e., approximately 1–1.5 h after lights turned on)^16^. The nest complexity was scored on a 6-point-scale using the protocol by Hess et al. (0: undisturbed nesting material, 1: disturbed nesting material, 2: flat nests, 3: cup, 4: incomplete dome, 5: complete dome; a detailed description can be found in the cited nest scoring protocol)^20,21^. Moreover, the location of the nests was noted for separated pair-housed mice to evaluate whether they prefer distance or proximity to the other mouse^16^. For this scoring, the cage compartments of the separated housing system were divided into six equally sized units.

### Burrowing

For testing the burrowing performance^22,23^, blue opaque plastic water bottles were used as burrows (7 cm × 7 cm × 11.5 cm, 3 cm diameter of bottle neck)^16^. The bottles were filled with approximately 140 ± 2 g food pellets normally supplied as diet^16,24,25^. After the mice had been transported in their home cages to the testing room and allowed to acclimatize for 30 min, they were transferred from their home cage to the testing cage (Polysulfone type III cages, 42 × 26 × 15 cm, approximately 0.5–1 cm bedding material, gridded cage lid, water bottle, feeder filled with a few pellets; Fig. 3), where they habituated for another 30 min. Then the burrow was placed parallel to the left long wall of the cage in the corner and the mice were monitored for two hours^16^. After the test, the amount of food pellets removed from the burrow was determined^16^. Since mice were tested simultaneously and therefore the position of the testing cages in the room slightly varied, the effect of the cage position was taken into consideration in data analysis.

### Anxiety-related behavior

The free exploratory paradigm^24,26^ was carried out in the testing cage to evaluate trait anxiety-related behavior^16^. The cage was opened, the burrow was removed from the cage, and a gridded cage lid (type I long, 34.5 cm × 14.5 cm) was attached to the back wall using wire so that the mice could climb on this novel object/ladder^16^. The mice were video monitored for 5 min^16^. The experimenters were present during the test and stood silently next to the cameras that were installed at a distance of approximately 1.5 m from the cages^16^. The latency to explore (i.e., the time until the mouse climbed onto the ladder with all four paws touching it for the first time) as well as the total duration of exploration were manually determined from the videos using an ethological analysis software (Etholog version 2.2.5; Ottoni 1999)^16^. The total duration of exploration was the sum of all exploration events. An exploration event started when the mouse touched the ladder with all four paws and ended when the mouse left the ladder. Walking along the edge of the cage was also considered as exploration, i.e., the exploration event did not end when the animal left the ladder and climbed onto the cage edge. Since mice were tested simultaneously and therefore the cage positions in the room slightly varied, the effect of the cage position was taken into consideration in data analysis.

### Ease of handling

During the weekly cage change in week 8 the ease of handling was examined^16^. The voluntary approach and interaction of the mice with the experimenter’s hand can give information on the “anxiety-related behavior in anticipation of handling”^16,27^. For the test, the cage was placed on a table and the cage top, the gridded lid, as well as the house or tunnel were removed^16^. The left gloved hand was placed in the cage opposite to the nest location, with the palm facing downwards^16^. In separated pair-housed mice, the test was performed simultaneously, i.e. the left hand was placed in the left compartment and the right hand in the right compartment^16^. During the 60-s testing period, the latency to first voluntary interaction with the experimenter’s hand, i.e., the contact of whiskers, nose, and/or paws with the hand, was determined and the interaction with the hand was scored (interaction score), as follows^16^:

- Score 0 = The mouse explored the experimenter’s hand by climbing on it.
- Score 1 = The mouse explored the experimenter’s hand by direct contact with the paws.
- Score 2 = The mouse explored the experimenter’s hand by direct contact with the whiskers and/or nose.
- Score 3 = The mouse carefully approached but did not touch the experimenter’s hand. Protected stretches towards the hand could be observed, i.e., the forepaws approach the hand and the hind paws stay in the same position.
- Score 4 = The mouse moved away from the hand and settled down at the largest possible distance. It made no attempts to approach the experimenter’s hand.

Subsequently, the animal was picked up by the tail and gently transferred to the back of the hand^16^. This process was scored on a 5-point scale (capture score, modified from Wahlsten et al.^28^):

- Score 0 = The experimenter caught the mouse at first attempt. The mouse showed minimal resistance to capture.
- Score 1 = The mouse escaped from the first capture attempt and completed less than one circuit of cage prior to capture.
- Score 2 = The mouse escaped from the first capture attempt and completed 1–2 circuits of cage prior to capture.
- Score 3 = The mouse escaped from the first capture attempt and completed more than 2 circuits of cage prior to capture. Occasionally, the mouse jumped onto the cage wall or the experimenter captured the mouse by the tail on the wall.
- Score 4 = The mouse jumped out of the cage.

Animals of this study were handled by tail since the non-aversive mouse handling techniques such as tunnel and cup handling had not been implemented in the animal facility at the time of study. However, since then, we introduced tunnel and cup handling to the facility and strongly recommend the use of gentle handling methods to minimize stress and anxiety in the mice^27,29^.

### Social interaction

In week 7 after the mice had been assigned to the housing systems, the approach-avoidance behavior of a ‘test’ mouse to an unfamiliar C57BL/6JRj female ‘target’ mouse was monitored in a 43.5 × 43.5 cm arena equipped with a perforated polycarbonate box (10 × 6.5 cm)^16^. Each mouse performed two 2.5-min sessions^16^. In the first 'no target' session, the ‘test’ mouse was allowed to explore the open arena freely with an empty perforated polycarbonate box^16^. In the second 'target' session, a ‘target’ mouse was placed into the perforated polycarbonate box, which allowed visual, olfactory, and acoustic interactions between the ‘test’ and ‘target’ mouse but prevented direct physical contact^16^. The ’target mice ‘ (*n* = 12) were only used for the purpose of this test, i.e., they were not part of the study groups to be tested. A video tracking software (Ethovision, Noldus, Netherlands) analyzed the distance the ‘test’ mouse moved in the arena as well as the time it spent in the ‘interaction zone around the target box (26.0 × 14.5 cm)^16^. The time spent in the interaction zone during the ‘target’ session was defined as social interaction^16^.

### Analysis of fecal corticosterone metabolites (FCMs)

Due to diurnal variations of FCM excretion^30^, feces were sampled at the same time of day (i.e., between approximately 8.30 and 11.30 A.M.)^16^. During this period, the mice had been individually housed in the testing cages (Makrolon type III cages, 42 × 26 × 15 cm) to investigate burrowing (and trait anxiety-related behavior)^16^. After this period, testing cages were stored at room temperature for 20–24 h before fecal pellets were collected from the cages using forceps. Fecal corticosterone metabolites (FCMs) were extracted and analyzed as previously described^30–32^.

### Analysis of hair hormones

Approximately 7.5 mg of hair were collected using an electric shaver for small animals (Aesculap Isis GT 420, Suhl, Germany) at the beginning and the end of the experiment, i.e., on the first and last day^16^. Both samples were taken from the same body location (back).

Hair corticosterone, progesterone, and dehydroepiandrosterone (DHEA) were analyzed [pg/mg] by liquid chromatography-mass spectrometry in the laboratory of Prof. Kirschbaum, Department of Psychology, Technische Universität Dresden, Germany, as described previously^33^.

### Statistical analysis

Statistical analysis was performed with IBM SPSS Version 25 (IBM Corporation, Armonk, NY, USA) and explorative data analysis and tests for normality (visual inspection of histograms and qq-plots, comparison of mean, standard deviation (sd) and median) were performed for each continuous parameter. Differences between the groups were analyzed using the linear mixed regression models with cage group and litter as random effects and the housing system as fixed factor. Post hoc Bonferroni comparisons were used to assess pairwise effects. Differences were considered significant at *p* < 0.05. Model diagnostics included visual inspection of residuals for normality. In most of the mixed regression models, the variance components of the random factors were negligible. Any exceptions from this model are listed below for each parameter.

### Nesting

Nest complexity scores were compared between single- and separated pair-housed mice only. Besides the housing system, the time as well as the interaction between time and housing system were included as fixed factors in the linear mixed regression models. Data from both time points were separately analyzed by comparing the 95% confidence (CI) intervals and means: if the mean value measured on a time point was not within the 95% CI of the other time point, the difference was considered significant. Differences between the time points were assessed by comparing the 95% confidence (CI) intervals and means, as described above.

### Burrowing

Besides the housing system, the time, the interaction between time and housing system, as well as the cage position were included as fixed factors in the linear mixed regression models. Data from both time points were separately analyzed by comparing the 95% confidence (CI) intervals and means: if the mean value measured on a time point was not within the 95% CI of the other time point, the difference was considered significant. Differences between the time points were assessed by comparing the 95% confidence (CI) intervals and means, as described above. The cage position was also included in the model to control for respective effects. Overall, one single- and one group-housed mice were excluded from statistics because they removed less than 5 g food pellets from the burrow (non-responders).

### Anxiety-related behavior

The cage position was also included as fixed effect in the model to control for respective effects.

### Ease of handling

Since the residuals of the variable “latency to the first voluntary interaction with the hand” were not normally distributed, the results of the Kruskal–Wallis-test were reported.

### Social interaction

The housing system, target, and the interaction between housing system and target were included as fixed factors in the linear mixed regression models to analyze the time spent in the interaction zone and the locomotor activity.

### Body weight

Random factors were also neglected for the analysis of body weight; a repeated measures ANOVA model was used instead.

### Fecal corticosterone metabolites (FCMs)

Besides the housing system, the time, and the interaction between time and housing system were included as fixed factors in the linear mixed regression models. Data from both time points were separately analyzed and differences between the time points were assessed by comparing the 95% confidence (CI) intervals and means, as described above. Overall, two single-housed mice, one group-housed mouse, and two pair-housed mice were excluded from statistics because there was not enough sample material for analysis.

### Analysis of hair hormone

Besides the housing system, the time as well as the interaction between time and housing system were included as fixed factors in the linear mixed regression models. Data from both time points were separately analyzed and differences between the time points were assessed by comparing the 95% confidence (CI) intervals and means, as described above. Two pair-housed mice were excluded from statistics because they there was not enough sample material for analysis.

## Results

### Nesting

Since the nests of groups and individuals have a varying shape and group-housed mice usually built a nest together, nest complexity scores were compared between single- and separated pair-housed mice only^16^. In the mixed regression model, neither time (F(1, 60) = 1.132, *p* = 0.292) nor the interaction between housing system and time (F(1, 60) = 1.649, *p* = 0.204) was significantly different, but the housing system significantly affected the nest complexity scores (F(1, 60) = 30.699, *p* < 0.001) with higher scores in pair-housed mice.

**Figure 4:**
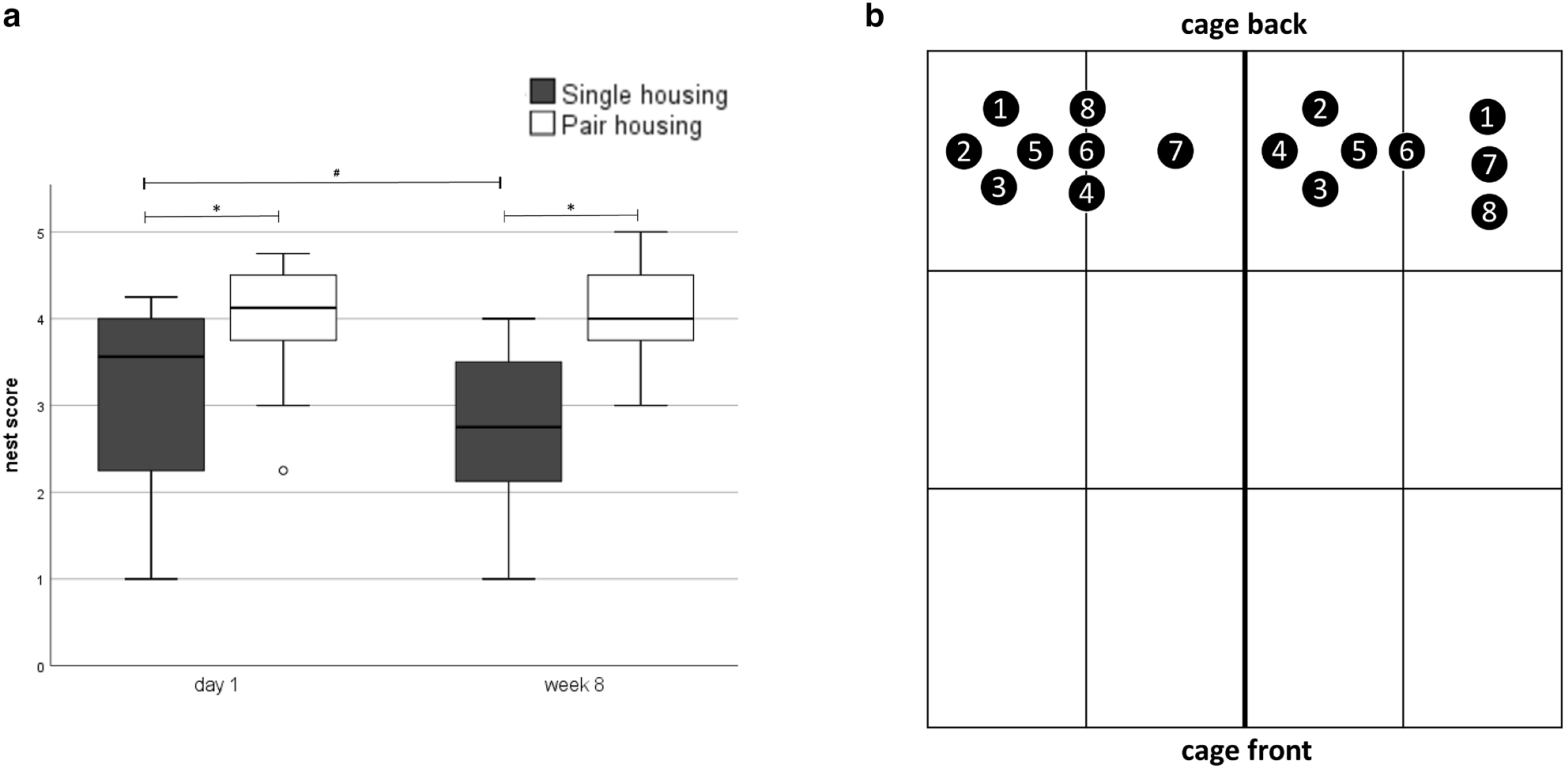
Nesting. (**a**) Nest complexity scores: Data are presented as boxplot diagrams: the box represents the interquartile range (IQR), box edges are the 25th and 75th quartile. The whiskers represent values which are not greater than 1.5 × IQR. Dots are outliers with values between 1.5‒3.0 × IQR. *p < 0.05 versus pair housing. Single housing: *n* = 16, pair housing: *n* = 16. (**b**) Nest positions in the pair housing system: the cage compartments were divided into six units. The numbers symbolize the unit/border where the nests were built. For nests of both mice of a pair, the same numbers were used (*n* = 8 pairs).

According to the 95% CI, nests of separated pair-housed mice were more complex compared to nests built by single-housed mice on day 1 (data are given as mean and 95% CI; single housing: 3.11 [2.68; 3–54]; separated pair housing: 4.02 [3.59; 4.44]) as well as in week 8 (single housing: 2.61 [2.18; 3.04]; separated pair housing: 4.06 [3.64; 4.49]) (Fig. 4a). Nest scores of single-housed mice were lower in week 8 when compared to day 1.

The groups achieved the following nest scores (IQR, median): 2.69–3.62, 3.50 on day 1; 3.13–4.00, 3.75 in week 8.

Figure 4b shows the position of nests built by separated pair-housed mice in week 8 after the transfer to the housing system. This parameter was of interest for the separated housing condition only to investigate whether they prefer resting in distance or proximity to the other mouse^16^. Since separated pair housed animals must be considered as a unit (*n* = 8), data were analyzed descriptively only^16^.

Overall, most mice built their nest under the food rack, which was located at the rear end of the cage^16^. One pair built nests with a distance of ≤ 1 unit from each other, four pairs with a distance of one unit, and three pairs with a distance of more than one unit.

### Burrowing

**Figure 5:**
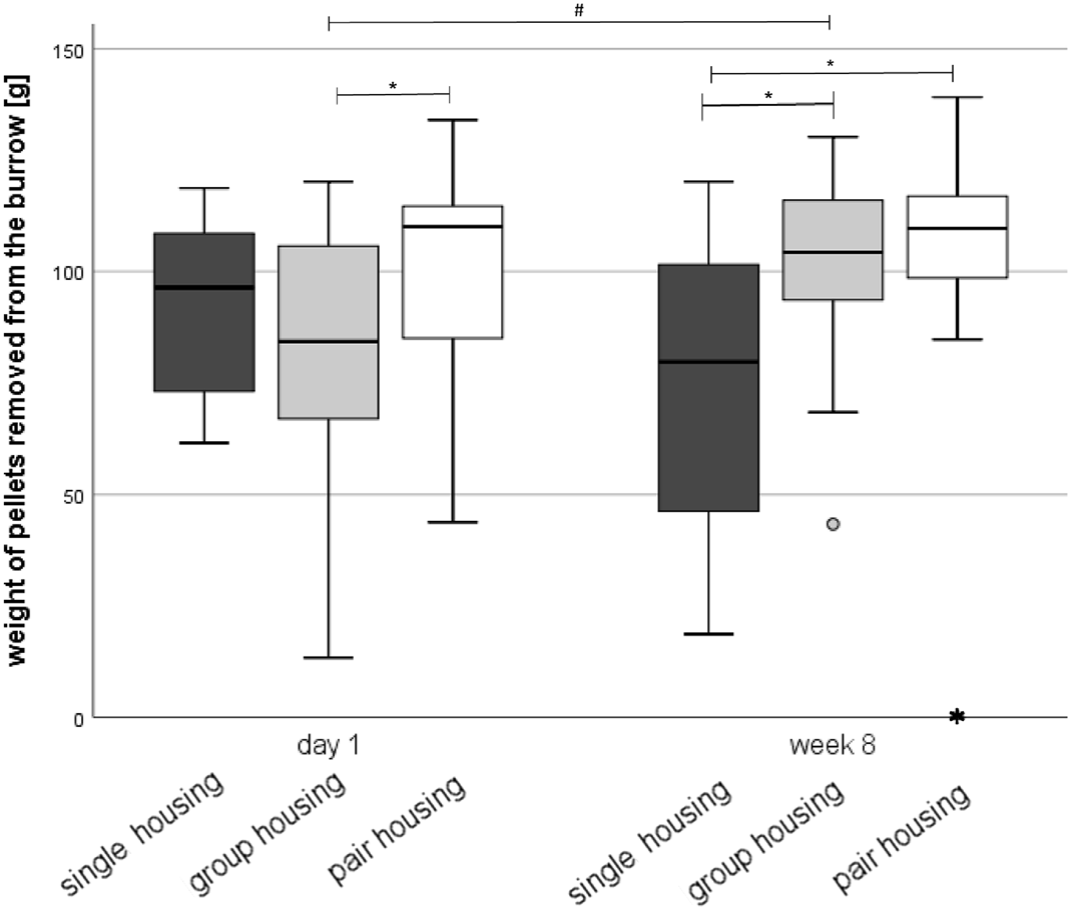
Burrowing performance. Data are presented as boxplot diagrams: the box represents the interquartile range (IQR), box edges are the 25th and 75th quartile. The whiskers represent values which are not greater than 1.5 × IQR. Dots are outliers with values between 1.5‒3.0 × IQR. Asterisks are outliers with values greater than 3.0 × IQR. Single housing: *n* = 15, group housing: *n* = 15, pair housing: *n* = 16. Overall, one single- and one group-housed mice were excluded from statistics because they removed less than 5 g food pellets from the burrow (non-responders). *p < 0.05 versus the other housing system; #*p* < 0.05 versus week 8.

The mixed regression models revealed that neither the cage position (F(3, 83) = 1.527, p = 0.214), time (F(1, 83) = 0.004, *p* = 0.951) nor the interaction between group and time (F(2, 83) = 3.074, *p* = 0.052) had an impact on the burrowing performance of the mice (Fig. 5), while the housing system (F(2, 83) = 3.125, *p* = 0.049) significantly affected the amount of pellets removed from the burrow. Post hoc Bonferroni analysis showed that the burrowing performance was increased in pair-housed mice when compared to single-housed mice (*p* = 0.04).

The 95% CI showed that the burrowing performance was higher in paired-housed mice than in group-housed mice on day 1 (single-housing: 85.11 [67.71; 102.51]; group-housing: 73.70 [56.62; 90.77]; separated pair housing: 99.05 [83.57; 114.54]). In week 8, single-housed mice (68.43 [51.03; 85.83]) removed less pellets from the burrow than group- (94.23 [77.16; 111.30]) and separated pair-housed (96.32 [80.84; 111.81]) mice. There was an increase over time in group-housed mice.

### Anxiety-related behavior

**Figure 6:**
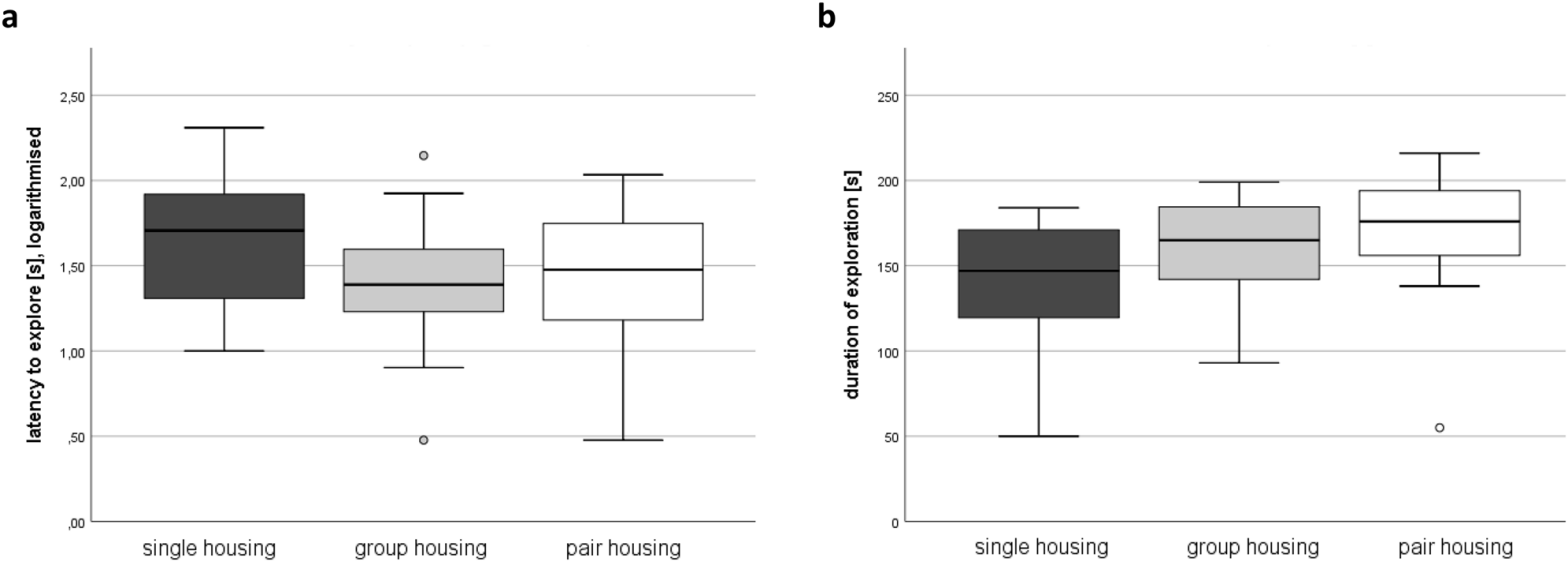
Anxiety-related behavior analyzed in the free exploratory paradigm. Latency to explore the gridded cage top (**a**) and total duration of exploration (**b**) in week 8. Data are presented as boxplot diagrams: the box represents the interquartile range (IQR), box edges are the 25th and 75th quartile. The whiskers represent values which are not greater than 1.5 × IQR. Dots are outliers with values between 1.5‒3.0 × IQR. Single housing: *n* = 16, group housing: *n* = 16, pair housing: *n* = 16.

The cage position in the testing room during the free exploratory paradigm did not affect the logarithm of the latency to explore (F(3, 27.2) = 1.015, *p* = 0.401) and the duration of exploration (F(3, 31.8) = 0.525, *p* = 0.668) in the respective mixed regression models. The models also revealed that the housing systems neither affected the logarithm of the latency to explore (F(2, 15.0) = 0.505, *p* = 0.614) nor the duration of exploration (F(2, 16.0) = 2.357, *p* = 0.127; Fig. 6).

### Ease of handling

**Figure 7:**
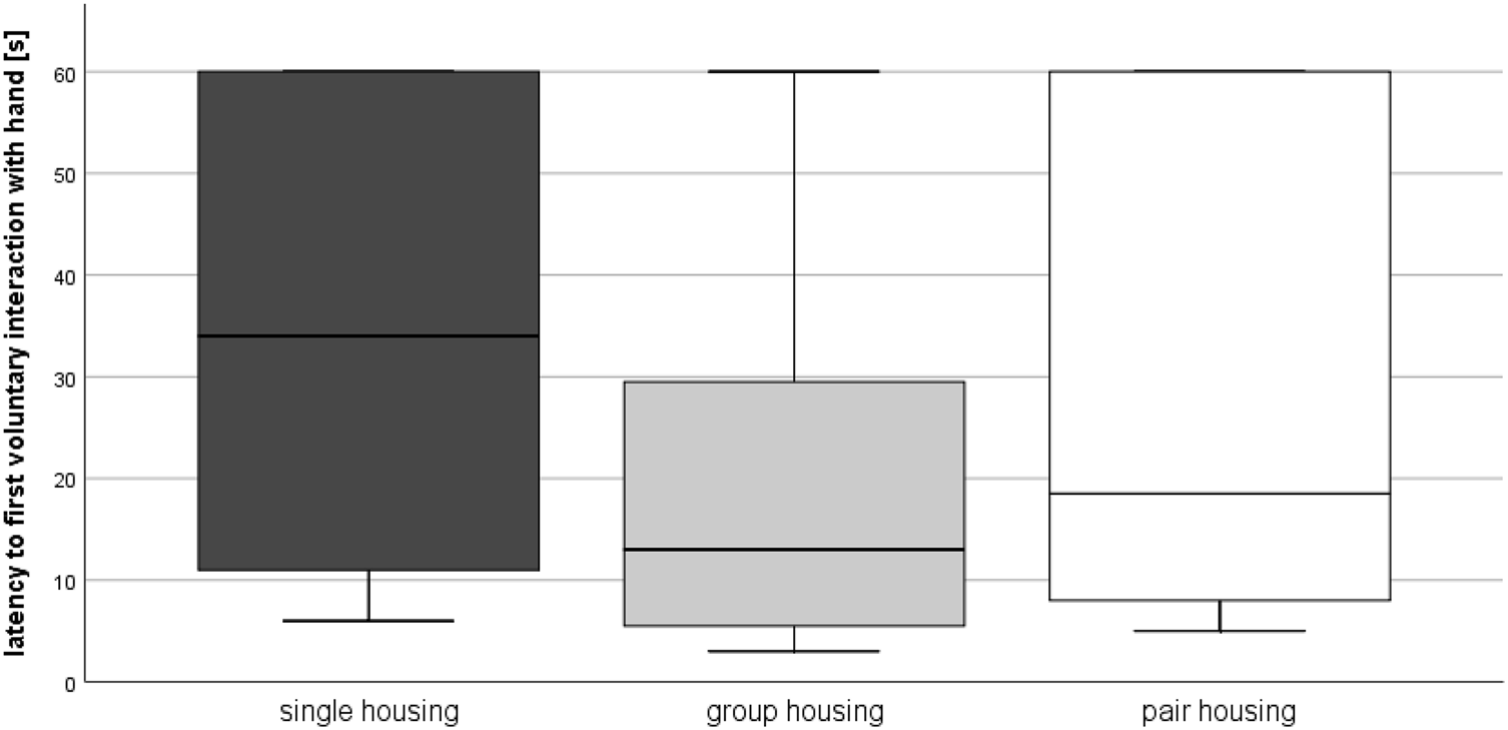
Ease of handling. Data are presented as boxplot diagrams: the box represents the interquartile range (IQR), box edges are the 25th and 75th quartile. The whiskers represent values which are not greater than 1.5 × IQR. Single housing: *n* = 16, group housing: *n* = 16, pair housing: *n* = 16.

After mice had lived for eight weeks in the respective housing systems, the ease of handling was analyzed. There were no significant differences in the latency to first voluntary interaction with the experimenter’s hand between the housing systems (Kruskal–Wallis-Test: Chi^2^ = 4.549, df = 2, *p* = 0.103; Fig. 7). However, the interaction scores significantly differed between the housing systems (F(2, 45) = 4.810, *p* = 0.013; Table 1), without any significant pairwise comparison. Moreover, the mixed regression model revealed (F(2, 286.7) = 4.485, *p* = 0.012) that group-housed mice (t = –2.038, *p* = 0.042) were easier to catch when compared to separated pair-housed mice (Table 1).

**Table 1:** Interaction and capture scores.

Housing condition	Interaction score	Capture score
Single housing	2 (2–3)	1 (0.25–2)
Group housing	2 (2–2)	0 (0–0)*
Separated pair housing	2 (2–2.75)	1 (0–2)

Data are given as median (25th quartile–75th quartile): *p < 0.05, versus separated pair housing. Single housing: *n* = 16, group housing: *n* = 16, separated pair housing: *n* = 16.

### Social interaction

**Figure 8:**
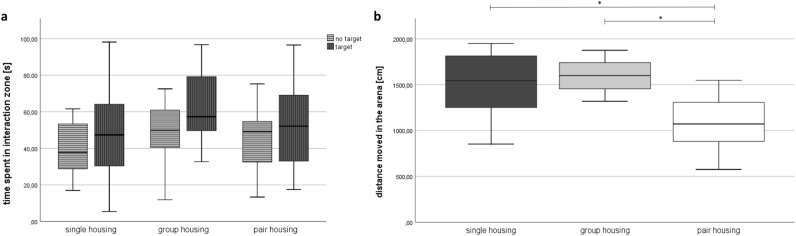
Social interaction test. (**a**) Time spent in interaction zone and (**b**) distance moved in the entire arena in absence of the target mouse in week 7. Data are presented as boxplot diagrams: the box represents the interquartile range (IQR), box edges are the 25th and 75th quartile. Single housing: *n* = 16, group housing: *n* = 16, pair housing: *n* = 16. *p < 0.05 versus the other housing system.

The time spent in the interaction zone was not affected by the housing condition (F(2, 14.8) = 1.638, *p* = 0.228) but by the presence or absence of the target mouse (F(1, 158.813) = 8.890, *p* = 0.004) (Fig. 8). Bonferroni post-hoc analysis revealed that the presence of a target mouse significantly increased the time spent in the interaction zone (*p* = 0.004). The interaction between housing system and target was not significant (F(2, 58.813) = 0.317; *p* = 0.730) The distance moved in the test-arena was significantly influenced by the housing system (F(2, 16.004) = 7.759; *p* = 0.004) with less locomotor activity in separated pair-housed mice compared to single-housed (Bonferroni post-hoc analysis: *p* = 0. 028) as well as group-housed mice (Bonferroni post-hoc analysis: *p* = 0.006). In the presence of the target mouse, the mice showed significantly less movement than in the absence of the target mouse (F(1,54.986) = 123.664, *p* < 0,001; Bonferroni post-hoc test *p* < 0.001). The interaction between group and target was significant (F(2, 54.986) = 5.015, *p* = 0.01). In contrast to other analyses, the random effect litter accounted for a substantial of the variance: 0.27 without target mouse, 0.14 with target mouse.

### Body weight

**Figure 9:**
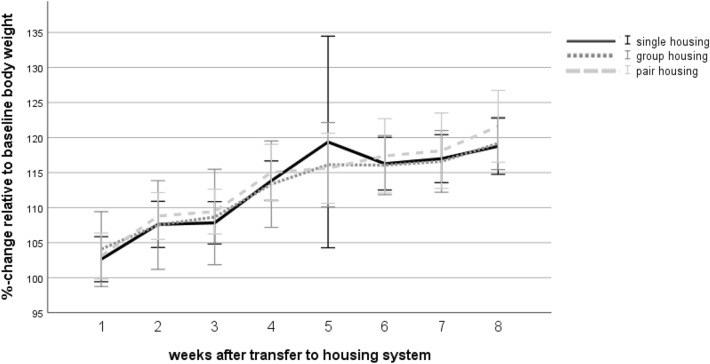
Body weight. Data are given as mean ± standard deviation. Single housing: *n* = 16, group housing: *n* = 16, pair housing: *n* = 16.

A repeated measures ANOVA with a Greenhouse–Geisser correction showed that mice put on weight over time (F(2.45, 110.40) = 102.84, *p* < 0.001). Tests of between-subject-effects revealed that the housing systems had no effect on the body weight gain (F (2, 45) = 0.25, *p* = 0.783; Fig. 9).

### Fecal corticosterone metabolites (FCMs)

In the mixed linear regression model with time, housing system, as well as interaction between housing system and time interaction as fixed effects, the interaction (F(2, 59.5) = 6.882, *p* = 0.002), the housing systems (F(2, 15.2) = 5.540, *p* = 0.016), and time (F(1, 59.5) = 9.466, *p* = 0.003) significantly affected FCM concentrations (Table 2). Bonferroni post-hoc analysis revealed that FCM concentrations were higher on day 1 than in week 8 (*p* = 0.003). FCM values were elevated in separated pair-housed mice when compared to single- (*p* = 0.017) as well as group-housed mice (*p* = 0.030).

**Table 2:** Fecal corticosterone metabolites.

Housing system	Day 1 (ng/0.05 g)	Week 8 (ng/0.05 g)
Single housing	53.98 ± 11.93*	57.02 ± 11.45
Group housing	60.58 ± 16.96*	52.91 ± 13.37
Separated pair housing	96.59 ± 46.82	60.71 ± 7.05**

Data are given as mean ± standard deviation. Fecal samples were analyzed from 14 single-housed, 15 group-housed, and 14 separated pair-housed mice. Overall, two single-housed mice, one group-housed mouse, and two pair-housed mice were excluded from statistics because there was not enough sample material for analysis.

*p < 0.05 versus separated pair-housing.

**p < 0.05 versus day 1.

The 95% CI revealed that there was an effect of the housing system on FCMs measured on day 1 with higher values in separated pair-housed mice when compared to single- as well as group-housed mice (single housing: 54.39 [41.17; 67.62]; group housing: 59.86 [45.98; 73.75]; separated pair housing: 59.56 [46.25; 72.86]). After eight weeks, the differences between the groups were not present anymore. In pair-housed mice, FCM concentrations decreased over time (week 8: 59.56 [46.25; 72.86]).

### Analysis of hair hormones

The mixed linear regression models with time, housing system, and interaction between time and housing system as fixed effects showed that the time significantly affected the hair corticosterone concentrations (F(1, 86) = 16.403, *p* < 0.001) with higher values in week 8, i.e., hair corticosterone increased over time. The housing system had no significant effects (F(2, 86) = 0.058, *p* = 0.44) and the interaction was not significant (F(2, 86) = 0.191, *p* = 0.826) (Table 3).

**Table 3:** Hair hormones.

Housing system	Baseline (pg/mg)	Week 8 (pg/mg)
**Corticosterone**		
Single housing	35.51 ± 7.24	40.44 ± 5.63
Group housing	34.87 ± 6.48	42.00 ± 9.56
Separated pair housing	34.58 ± 9.16	40.53 ± 6.72
**Progesterone**		
Single housing	0.50 ± 0.07	0.99 ± 0.08*
Group housing	0.53 ± 0.10	1.28 ± 0.52
Separated pair housing	0.55 ± 0.08	0.93 ± 0.31*
**Dehydroepiandrosterone (DHEA)**		
Single housing	6.29 ± 0.48	3.11 ± 0.66
Group housing	6.19 ± 0.34	3.36 ± 0.68
Separated pair housing	6.30 ± 0.69	2.70 ± 0.40*

Data are given as mean ± standard deviation. Hair samples were analyzed from 16 single-housed, 16 group-housed, and 14 separated pair-housed mice. Two pair-housed mice were excluded from statistics because they there was not enough sample material for analysis.

*p < 0.05 versus group-housing.

For hair progesterone concentrations, there were significant effects of time (F(1, 86) = 104.569, *p* < 0.001) with higher values in week 8, housing system (F(2, 86) = 3.369, *p* = 0.039), as well as interaction between the housing system and time (F(2, 86) = 4.087, *p* = 0.020). Bonferroni post-hoc analysis revealed that progesterone concentrations were higher in group-housed mice than pair-housed mice (*p* = 0.026) (Table 3). In week 8, the 95% CI indicated that group housing elevated progesterone values when compared to single and separated pair-housing (single housing: 0.99 [0.86; 1.13]; group housing: 1.28 [1.15; 1.41]; separated pair housing: 0.93 [0.79; 1.07]).

There was a significant effect of the interaction between the housing system and time (F(2, 86) = 3.455, 0.036), the housing system (F(2, 86) = 1.851, *p* = 0.163), and time (F(2, 86) = 746.871, *p* < 0.001) on DHEA concentrations. At baseline, higher values were measured than in week 8 (Table 3). According to the 95% CI, group housing increased DHEA levels when compared to separated pair-housing in week 8 (group housing: 3.36 [3.08; 3.63], separated pair housing: 2.70 [2.40; 3.00]).

## Discussion

Although group housing should be the gold standard for female laboratory mice, some experimental settings require the animals to be kept individually, which may negatively affect their well-being. To refine individual housing, we introduced the separated pair housing system for mice and compared it with single as well as group housing. We systematically evaluated the effects of these housing systems on a range of behavioral and physiological parameters to assess the well-being of female C57BL/6JRj mice: nesting, burrowing behavior, trait anxiety-related behavior, the ease of handling, social behavior, body weight, and hormone (metabolites) concentrations in feces and hair were determined.

The main short-term effects of separated pair-housing were that transferring the mice to the separated pair housing system increased FCM concentrations on day 1 and resulted in higher complexity nests when compared to single-housed mice. While acute stress levels returned to the levels of single- and group-housed mice in week 8, pair-housing had clear positive long-term effects on nesting behavior. In week 8, nests still achieved higher scores than those of single-housed mice. Another long-term effect was reduced locomotor activity of separated pair-housed mice in a novel test arena compared to mice from the other housing systems. Independent of the time point, pair housing improved the burrowing performance. Moreover, mice kept in the separated pair housing system were more difficult to catch than group-housed animals, but they were no more difficult to handle than single-housed mice. Separated pair housing did not significantly affect burrowing behavior, trait anxiety-related behavior, voluntary interaction with the experimenter’s hand, and body weight. Independent of the housing system, hair corticosterone, progesterone, and dehydroepiandrosterone concentrations changed with age.

To determine stress levels, hormone (metabolites) concentrations were analyzed in feces and hair. While FCMs reflected the hypothalamic–pituitary–adrenal (HPA) axis activity of the past night on day 1 or in week 8 after transfer to the housing systems^30^, hair hormones were used as retrospective biomarkers^33,34^. According to latest findings, hair corticosterone may not mirror the stress levels the animals experienced during the entire experiment. In male Sprague–Dawley rats, it was demonstrated that hair glucocorticoids rather reflected recent or ongoing stress^35^. Our FCMs analysis indicated that female mice experienced more short-term stress during the first night in the separated pair housing system than in the single housing system. This may be due to the new environment and/or the unfamiliar female on the other side of the cage divider. It remains unclear whether the elevated HPA axis activity derived from positive or negative affective states. It is also possible that differences in the IVC systems caused the changes in FCM concentrations. For example, the cage ventilation rate was higher in the separated pair housing system (75 changes per hour) than in IVCs used for single and group housing (50–60 changes per hour), which can cause unfamiliar noises, olfactory stimuli, and heat loss through convection. Since cages used for single and group housing were ventilated by the same system, animals assigned to these study groups had already been familiar with the noises and cage ventilation. After eight weeks, FCM concentrations returned to normal in separated pair-housed mice, indicating social habituation to the other mouse or the IVC system. We assume that FCM concentrations had already decreased within the first days or weeks in the new housing system, however, our study design did not include an earlier time point for FCM analysis.

In contrast to FCM, hair corticosterone concentrations were not affected by pair housing. DHEA concentrations showed an opposite trend in the present study (i.e., a decrease from day 1 to week 8). Elmi et al.^36^ found a mild positive correlation of age and DHEA concentrations in male mice. This discrepancy may be due to sex differences. In contrast to humans, DHEA is not produced by the adrenal glands, but rather by the brain in rodents^37,38^. It was shown that DHEA is regulated by hormones that are secreted in response to stress stimuli: the administration of corticotropin releasing hormone (CRH) and adrenocorticotropic hormone (ACTH) elevated brain and plasma concentrations of DHEA in female and male rats^39^. Elmi et al. reported that hair DHEA concentrations were higher in male group-housed mice than in those kept as pairs with a female, which may indicate higher social interaction^36^. This phenomenon was also observed in our study although statistical significance was not reached.

Nesting and burrowing can give valuable information on the well-being of mice. These behaviors are reduced when essential needs are not met^40^. Nesting behavior suggested that separated pair housing fostered the well-being of female mice when compared to single housing on day 1 and in week 8. However, this phenomenon may also be attributed to the differences in the ventilation rates^41,42^. Interestingly, single housing deteriorated nest complexity scores over time, which may indicate impaired well-being. With regard to the burrowing performance, pair housing appeared to improve well-being of the mice regardless of the time of testing. In week 8, single-housed mice tended to burrow fewer pellets from the bottle than mice from the other housing systems. In contrast to group housed mice, which burrowed more food pellets from the bottle in week 8 than on day 1, the burrowing performance decreased over time in single-housed mice, which may be associated with long-term harmful effects of social isolation on the animals’ well-being. In our previous study, burrowing behavior of male C57BL/6JRj mice did not differ between the housing groups in week 8^16^.

Similar to the observations we made in male C57BL/6JRj mice^16^, we found that female separated pair-housed mice moved less than single- and group-housed females in the arena of the social interaction test when an intruder was absent. Their locomotor activity was also reduced when compared to group-housed mice in the presence of an unfamiliar mouse. A decrease in exploratory behavior may be caused by stress^43–45^, though neither FCM nor hair corticosterone analysis indicated that separated pair-housed mice suffered from higher stress levels in week 8. Since the floor area of the cage compartments, in which the separated pair-housed mice were kept (527 cm^2^), varied only slightly from the floor area of the Polysulfone type I long (416 cm^2^) and type II long (544 cm^2^) cages, we did not expect the floor area of the home cages to have influenced the locomotor activity in the social interaction test. Future analysis of home cage activity could give better insights into the underlying reasons for this observation. Due to the reduced exploratory behavior of separated pair-housed mice, the time spent in the interaction zone during the social interaction test and the free exploratory paradigm must be interpreted with caution.

Although social isolation can affect anxiety-related behavior, depending on mouse strain, age, and duration of isolation^46,47^, trait anxiety in the free exploratory paradigm did not differ between the three housing systems. As previously observed in males, separated pair-housed mice were more difficult to catch in their home cages than group-housed mice^16^. They may be less used to other moving objects in their environment and the experimenter’s hand may have triggered their flight response. Another potentially responsible aspect was discussed in our previous study^16^: In contrast to cages used for single and group housing (i.e., Polysulfone type I and II long), the food unit of separated pair housing cages (i.e., Green Line IVC Sealsafe PLUS Rat) remained on top of the cages when the animals were picked from the cages for routine husbandry procedures^16^. The animal care technicians reported that they were usually hiding under the food unit and clinging tightly to the grid^16^. If more force had to be applied to detach them from the grid than picking up a mouse from the cage floor, separated pair-housed mice may have had worse handling experiences than animals from the other housing systems and therefore are more inclined to flee from the hand^16^.

Interestingly, hair corticosterone concentrations increased over time independent of the housing system. The same effect was found in our previous study in male mice^16^. An increase over time in hair corticosterone was also observed in wild-derived house mice (Mus musculus domesticus) that were born in the laboratory and housed under semi-natural conditions over a prolonged period^48^. In contrast, Elmi et al. reported that hair corticosterone levels decreased with age in male C57BL/6 J and C57BL/6OlaHsd^36^. This discrepancy may be explained by differences in housing and husbandry procedures, i.e., a semi-natural environment potentially provided a broader range of enrichment increasing the arousal state of the animals. While Elmi et al. used tunnel and cup handling to pick mice up, mice in the present study were handled by tail, which can elevate (stress-induced) plasma corticosterone levels^49,50^. Therefore, we hypothesize that tail handling may have resulted in higher hair corticosterone concentrations. To our knowledge, the effects of handling-techniques on hair corticosterone levels have not been investigated to date.

Taken together, the results of our study demonstrated that the transfer to the separated pair housing system caused short-term stress in female C57BL/6JRj mice, while long-term effects of separated pair housing were ambiguous. The increase in nesting and burrowing behavior suggested that separated pair housing may be beneficial to long-term single housing. However, the decrease in locomotor activity was not in line with this. Moreover, the burrowing performance over time indicated that group housing—the gold standard for housing female laboratory mice—may foster well-being. The study results emphasized how important it is to consider and report the housing conditions as well as their effects on biochemical and behavioral parameters in animal-based research in order to improve reproducibility.

## Supplementary Information

Supplementary Information.
